# Prolonged Hypoglycemic Encephalopathy: A Case Report

**DOI:** 10.1002/ccr3.9711

**Published:** 2024-12-06

**Authors:** Newton Ashish Shah, Santosh Bastola, Abhishek Kumar Shah, Manish Yadav

**Affiliations:** ^1^ Institute of Medicine Tribhuwan University Teaching Hospital Kathmandu Nepal; ^2^ Department of Emergency Medicine Tribhuwan University Teaching Hospital Kathmandu Nepal; ^3^ Maharajgunj Medical Campus Institute of Medicine Kathmandu Nepal

**Keywords:** diabetes, encephalopathy, endocrinology, hypoglycemia

## Abstract

Hypoglycemic encephalopathy is a rare yet life‐threatening diabetes complication, where overlapping symptoms with neurological conditions can delay diagnosis. Early recognition and treatment are crucial to prevent irreversible brain damage. Clinicians should suspect hypoglycemia in diabetic patients with altered mental status, as timely intervention improves outcomes and reduces mortality.

## 1 Introduction

Hypoglycemic encephalopathy (HE) refers to a condition where low blood glucose levels cause metabolic changes, in the brain [1]. The common cause is type 2 diabetes mellitus with overdoses of oral hypoglycemic medications [2]. Immediate intake of glucose can help relieve acute symptoms like fatigue, nausea, difficulty concentrating, and vision [3]. If left untreated, hypoglycemia can lead to outcomes such as memory loss, temporary motor impairments, a persistent vegetative state, and even death in a percentage of cases [4, 5, 6, 7].

Diagnosing encephalopathy can be challenging because of a lack of awareness about the condition and an incomplete medical history regarding medication use and symptom onset timing. The range of findings is varied and often nonspecific, sometimes causing confusion with ischemic stroke because of similar acute symptoms and imaging results [8]. Delays in treatment may prolong encephalopathy with long‐term consequences [9].

This case study details the experience of a 61‐year man, with type 2 diabetes mellitus who suffered from hypoglycemia leading to severe HE and eventual failure of multiple organs. The instance highlights the significance of diagnosing and handling hypoglycemia in individuals, with diabetes showcasing the issues that may result from delayed care. Through sharing this case our goal is to stress the importance of observation and immediate action to avert consequences, in comparable situations.

## 2 Case History/Examination

A 61‐year‐old male with type 2 diabetes and systemic hypertension was brought to the emergency department with a decreased level of consciousness of unknown duration. The patient was last seen conscious at 6 p.m. the previous evening, after which he went to bed following a meal and medication. The patient was found unconscious at 2 a.m., 6 hours before being brought to the hospital. Two recent falls were also noted, first one 2 days prior and another in the morning before this event. There were no symptoms such as fever, abnormal body movements, chest pain, cough, rashes, decreased urine output, or substance use.

Upon arrival, the patient was unconscious with a snoring airway. Respiratory rate was 14 breaths per minute, oxygen saturation 98% on room air, pulse rate 68 beats per minute, and blood pressure 140/70 mmHg. Neurologically, the Glasgow Coma Scale (GCS) score was 7 (E1V2M4), with 2 mm pupils bilaterally and sluggish reaction to light. Body temperature was 97°F.

## 3 Differential Diagnosis, Investigations, and Treatment

A rapid glucose test revealed a critically low level of 19 mg/dL. An electrocardiography, arterial blood gas (ABG) measurement, and urinary catheterization were done following a bolus of 50 mL of 50% dextrose that corrected the glucose level. An extended focused assessment with sonography for trauma (EFAST) was performed and found unremarkable. The patient was triaged as Red as indication of life‐threatening urgency and moved to the resuscitation area.

In resuscitation, the patient's airway was obstructed because of snoring, but there were no secretions or blood. Vitals showed a pulse rate of 72 bpm, blood pressure of 140/70 mmHg and respiratory rate of 16 breaths per minute, with oxygen saturation at 92% on room air. Decreased air entry and crepitations were noted on auscultation. The GCS remained 7, with pupils sluggishly reactive at 2.5 mm bilaterally. A thorough head‐to‐toe examination revealed no additional abnormalities. Neurological examination showed sluggish pupils, extensor plantar reflexes bilaterally, and hypoactive deep tendon reflexes. A non‐contrast CT (NCCT) of the head was performed to rule out intracranial pathology.

**FIGURE 1:**
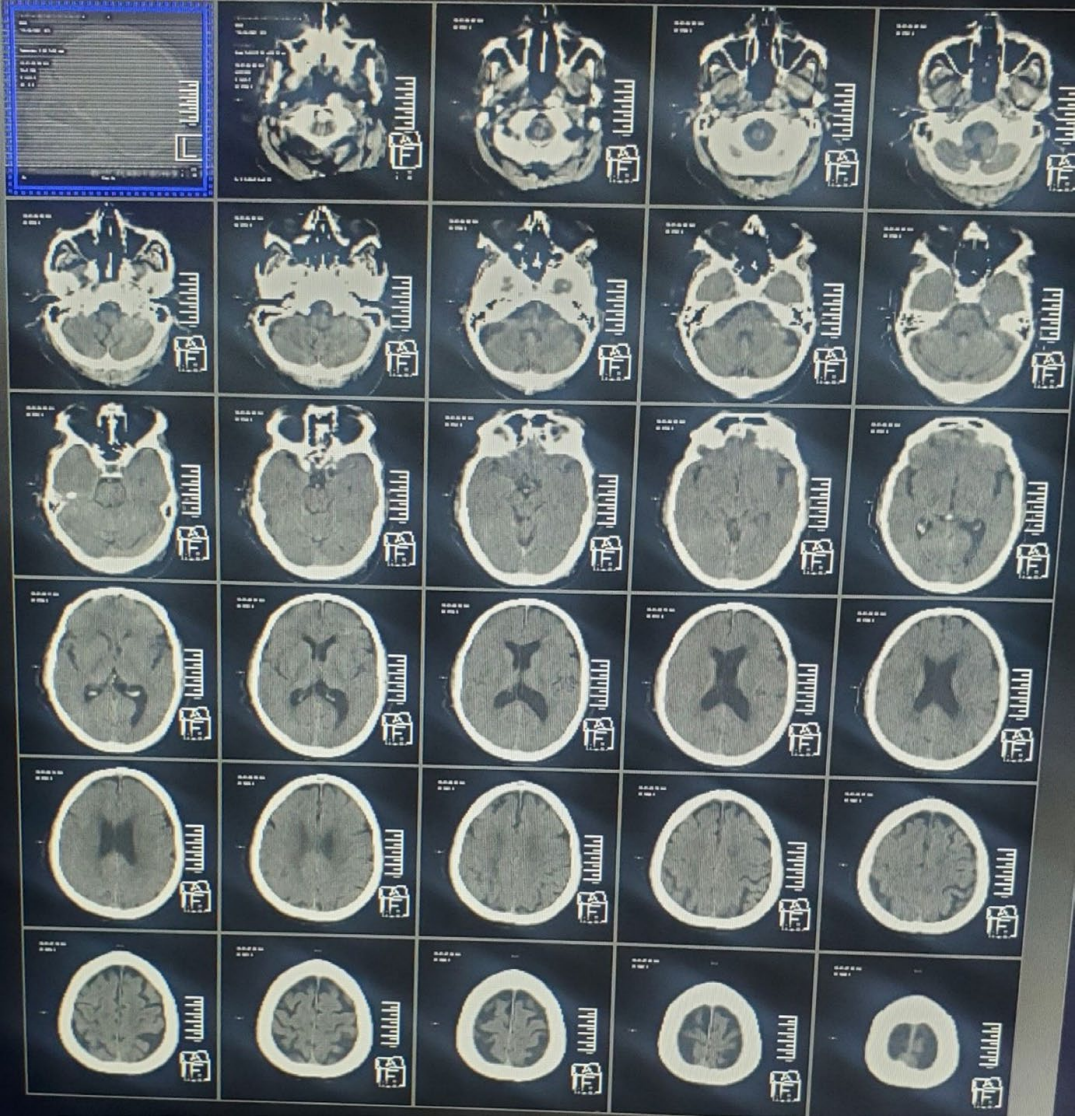
CT head showed no evidence of ischemic changes, intracranial hemorrhage, and space‐occupying lesions.

Postresuscitation, the glucose levels normalized (random blood sugar: 110 mg/dL), but the patient remained comatose (GCS: E1V1M4). Neurological reassessment showed no meningeal signs. ABG analysis indicated compensated metabolic acidosis. The patient was intubated and placed on mechanical ventilation. Intravenous antibiotics and maintenance fluids were administered. Laboratory tests, including complete blood counts, serum electrolytes, and renal function tests, were normal. A urine toxicology screen was negative. The CT head showed no ischemic changes, hemorrhage, or space‐occupying lesions (Figure 1). HE was suspected because of the initial low glucose and prolonged hypoglycemia. The patient was transferred to the intensive care unit (ICU) for further management.

**FIGURE 2:**
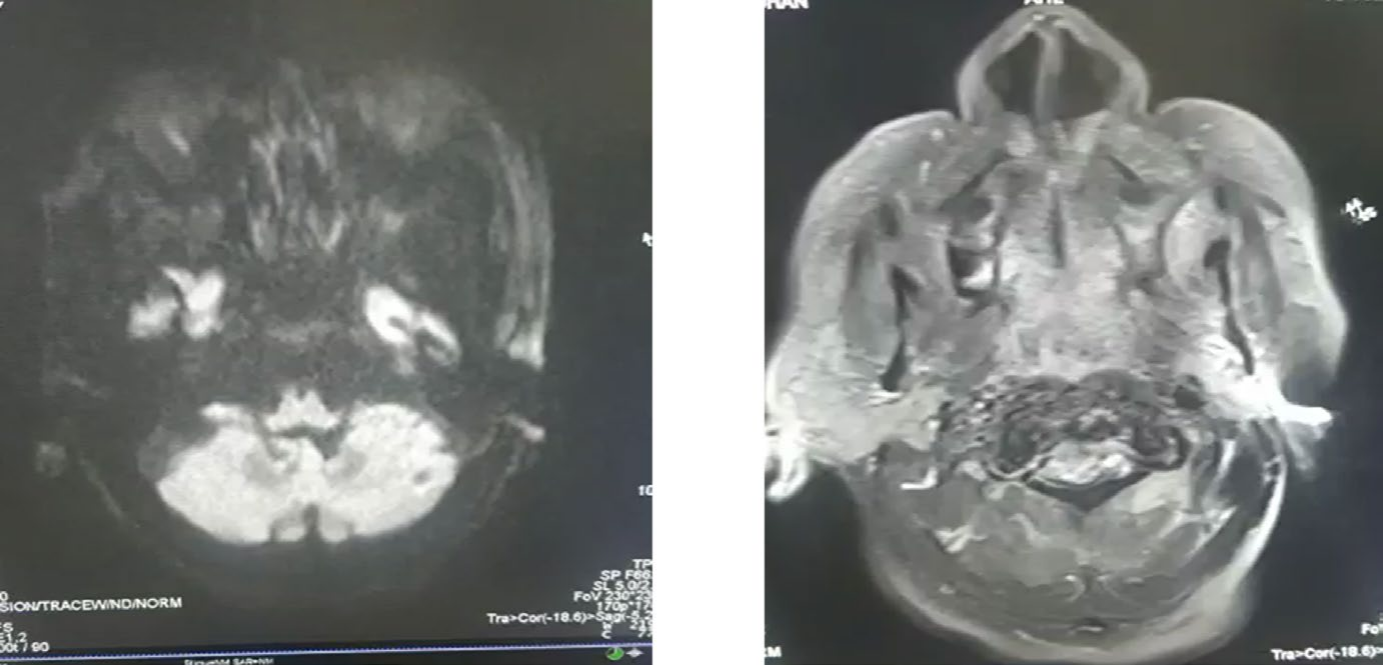
MRI of the brain showed signal alterations with restricted diffusion in the bilateral cerebral cortex, basal ganglia, and caudate nucleus, indicative of ischemia and cortical necrosis, likely resulting from hypoxic–ischemic injury.

In the ICU, the patient continued ventilatory support because of persistent low sensorium. A lumbar puncture revealed normal cerebrospinal fluid (CSF) findings. Repeat CT showed no new changes. Brain MRI revealed signal changes with restricted diffusion in the bilateral cerebral cortex, basal ganglia, and caudate nucleus, suggesting ischemia with cortical necrosis, likely due to hypoxic–ischemic injury (Figure 2). Differential diagnoses included toxic encephalopathy.

An elective tracheostomy was performed to facilitate prolonged ventilation. The patient was treated with IV antibiotics according to culture sensitivity results, antiplatelet agents, anticonvulsants, and supportive care including physiotherapy.

## 4 Conclusion and Results

Despite normalization of blood glucose levels, the patient showed no improvement in sensorium. The patient's condition further deteriorated, developing sepsis, which progressed to multi‐organ failure. Unfortunately, after 25 days of hospital stay, the patient succumbed to his illness.

## 5 Discussion

Although currently ill‐defined, HE, which occurs during deep and/or prolonged hypoglycemia, is a sustained comatose state. Seizures and other neurological deficits may also be observed. The short‐ and long‐term evolution of HE, from full recovery to a persistent vegetative state, remains yet poorly understood [10, 11, 12]. In this case, a 61‐year‐old male with type 2 diabetes mellitus experienced prolonged hypoglycemia, resulting in severe HE and multi‐organ failure, despite prompt medical intervention. This case underscores the critical importance of early diagnosis and treatment to prevent irreversible damage.

The exact mechanism of tissue damage in HE is not fully understood. It is postulated that hypoglycemia disrupts the Krebs cycle, leading to increased oxaloacetate production from aspartic acid, which contributes to tissue apoptosis and necrosis [13]. This selective vulnerability primarily affects high‐energy‐demanding areas of the brain, such as the cerebral cortex, hippocampus, basal ganglia, and cerebellum [14]. In our patient, MRI revealed signal changes with restricted diffusion in those regions as shown in Figure 2.

In the clinical context, HE presents a diagnostic challenge because of its overlapping symptoms with other neurological conditions, such as acute ischemic stroke. Our patient's initial presentation with decreased consciousness, unresponsiveness, and focal neurological deficits raised concerns for a stroke, but imaging ruled out ischemic changes. This case highlights the importance of considering hypoglycemia in the differential diagnosis, especially in diabetic patients, to avoid delays in treatment.

Restricted diffusion in the MRI in various regions in brain as mentioned above is suggestive of HE in most cases [3]. Neuropathologic studies have demonstrated that the (involvement of gray matter) cerebral cortex, hippocampus, and BG are commonly affected sites in severe hypoglycemia; however, the white matter is usually spared [15]. But recent studies have shown that white matter was more sensitive to hypoglycemia than previously thought and there was no specific association between the patterns of injury and clinical outcomes whether the cerebral cortex, deep gray matter, and/or white matter were affected. There can be involvement of both gray and white matter, selective involvement of gray matter, and selective involvement of white matter [16].

HE is similar to tissue ischaemia in causing reversible cytotoxic oedema mainly in the cerebral cortex and deep gray matter including the globus pallidus and thalami and white matter involvement is seen only in later stages [17]. Several different conditions, including seizure, drug toxicity, viral encephalitis, and metabolic encephalopathy, have reversible diffusion restrictions similar to those in hypoglycemia [18]. However, most of these conditions are associated with other specific abnormalities. Therefore, a diagnosis of HE is not difficult to distinguish from several other conditions, even if the conditions show reversible diffusion abnormalities on MR imaging [3].

However, there is a very poor correlation between blood glucose levels and imaging findings. Also, MRI findings do not carry relevance to long‐term prognosis. The time course of the disease and the findings across multiple vascular territories may help make this differentiation.

The prognosis of HE is influenced by several factors, including the severity and duration of hypoglycemia [5]. In this case, the patient's prolonged hypoglycemia, combined with his diabetic condition, were significantly poor prognostic indicators. Studies suggest that hypoglycemia lasting longer than 8 hours is associated with a worse outcome, which aligns with our patient's lack of improvement in sensorium despite glucose correction [9]. Hypoglycemia associated with Diabetes carries a worse prognosis as compared with other causes.

Furthermore, the MRI findings, although crucial in assessing brain involvement, did not strongly correlate with the patient's clinical outcome, indicating that imaging results alone may not be reliable predictors of prognosis. However, normal DWI scans on initial presentation have been shown to predict a good prognosis. The presence of seizures at diagnosis has been shown to correlate with poor outcomes in some studies, but results have been variable in other trials [11].

Management of HE involves rapid glucose administration and supportive care [3]. Our patient received immediate glucose correction and was intubated because of persistent low sensorium. Despite these interventions, the patient's condition deteriorated, leading to sepsis and multi‐organ failure. This outcome emphasizes the challenges in managing prolonged hypoglycemia and the need for continuous monitoring and aggressive treatment to mitigate the risks of severe complications.

Previous studies have shown that prolonged HE is a severe condition leading to a poor long‐term outcome [9]. Our case adds to the understanding that delayed recognition and prolonged hypoglycemia, especially in diabetic patients, are associated with poorer outcomes. It also reinforces the need for heightened vigilance in monitoring diabetic patients, particularly those on hypoglycemic agents, to prevent such catastrophic events.

This case illustrates the devastating consequences of delayed recognition and treatment of hypoglycemia in diabetic patients. It serves as a reminder for clinicians to maintain a high index of suspicion for hypoglycemia in cases of altered mental status, particularly in diabetic individuals. Early intervention is crucial to prevent irreversible brain injury and improve patient outcomes. Continuous monitoring and aggressive management are essential to mitigate the risks of severe complications in such cases.

## Author Contributions

**Newton Ashish Shah:** conceptualization, data curation, supervision, writing – original draft, writing – review and editing. **Santosh Bastola:** conceptualization, supervision, writing – review and editing. **Abhishek Kumar Shah:** writing – original draft. **Manish Yadav:** writing – review and editing.

## Ethics Statement

The authors have nothing to report.

## Consent

Written informed consent was taken from the patient before writing the case report which can be made available upon Journal request.

## Conflicts of Interest

The authors declare no conflicts of interest.

## Data Availability

The authors have nothing to report.
